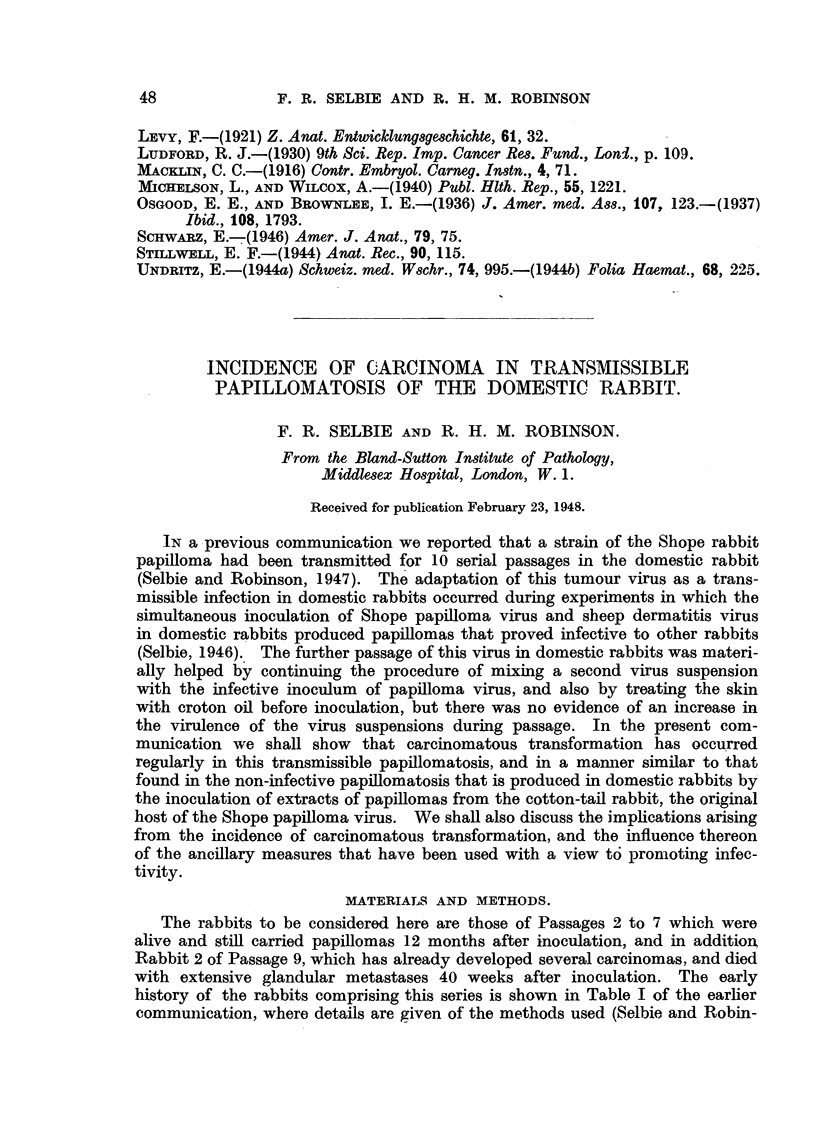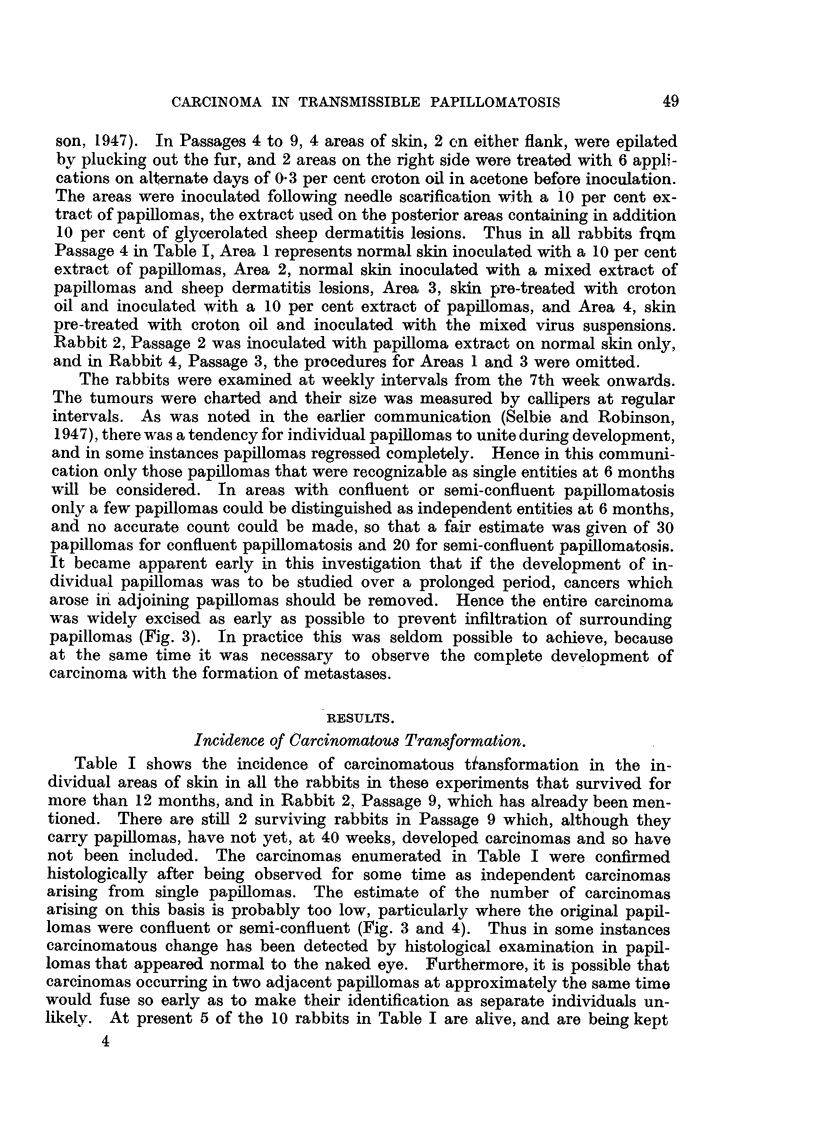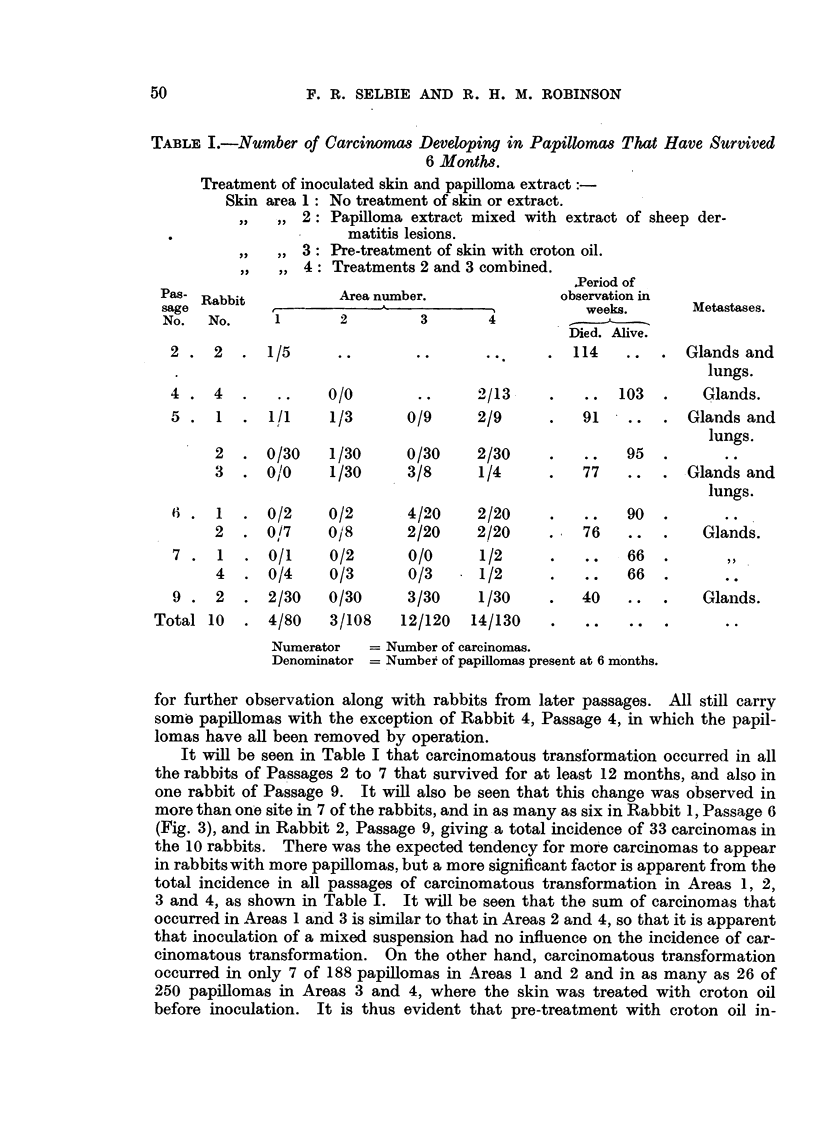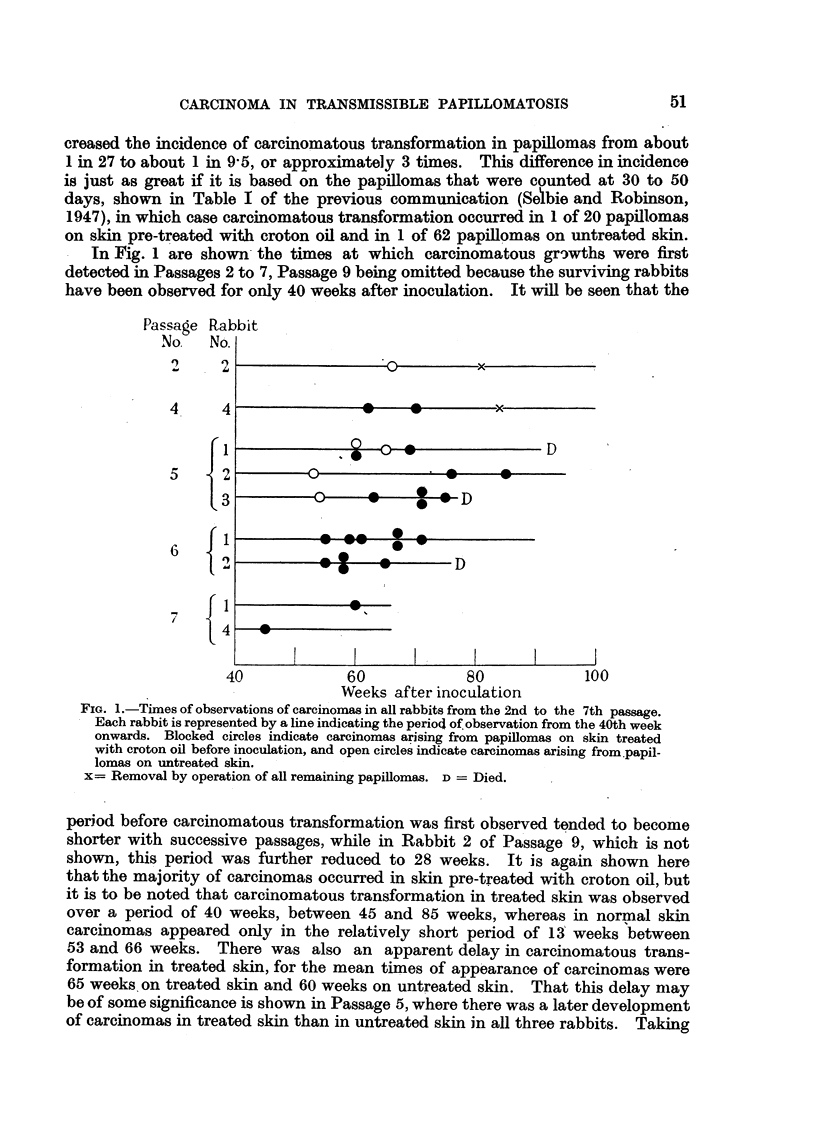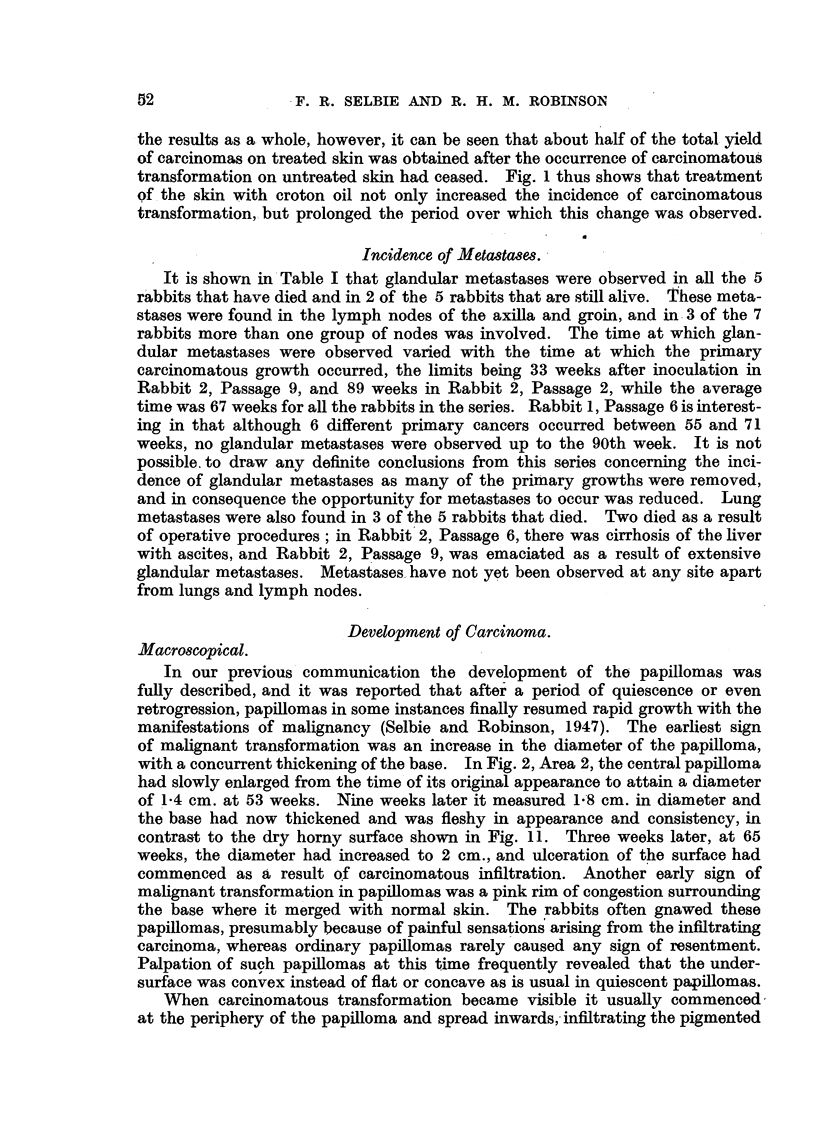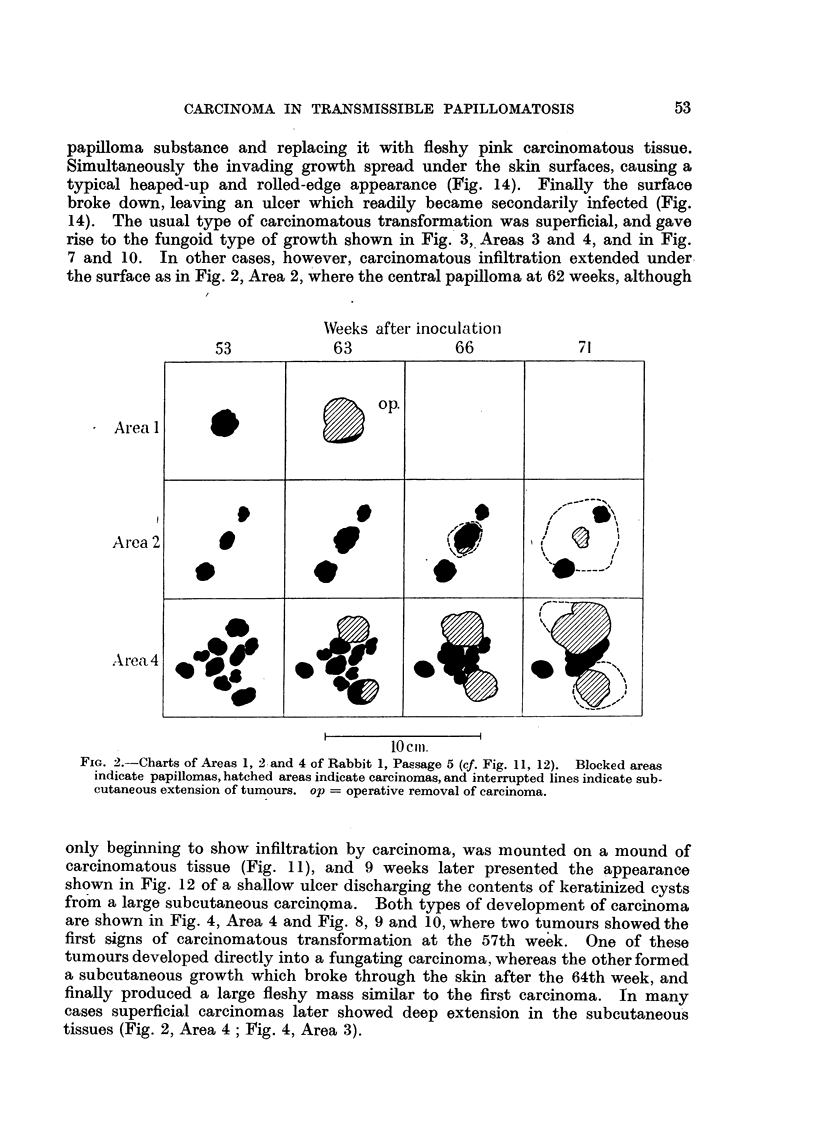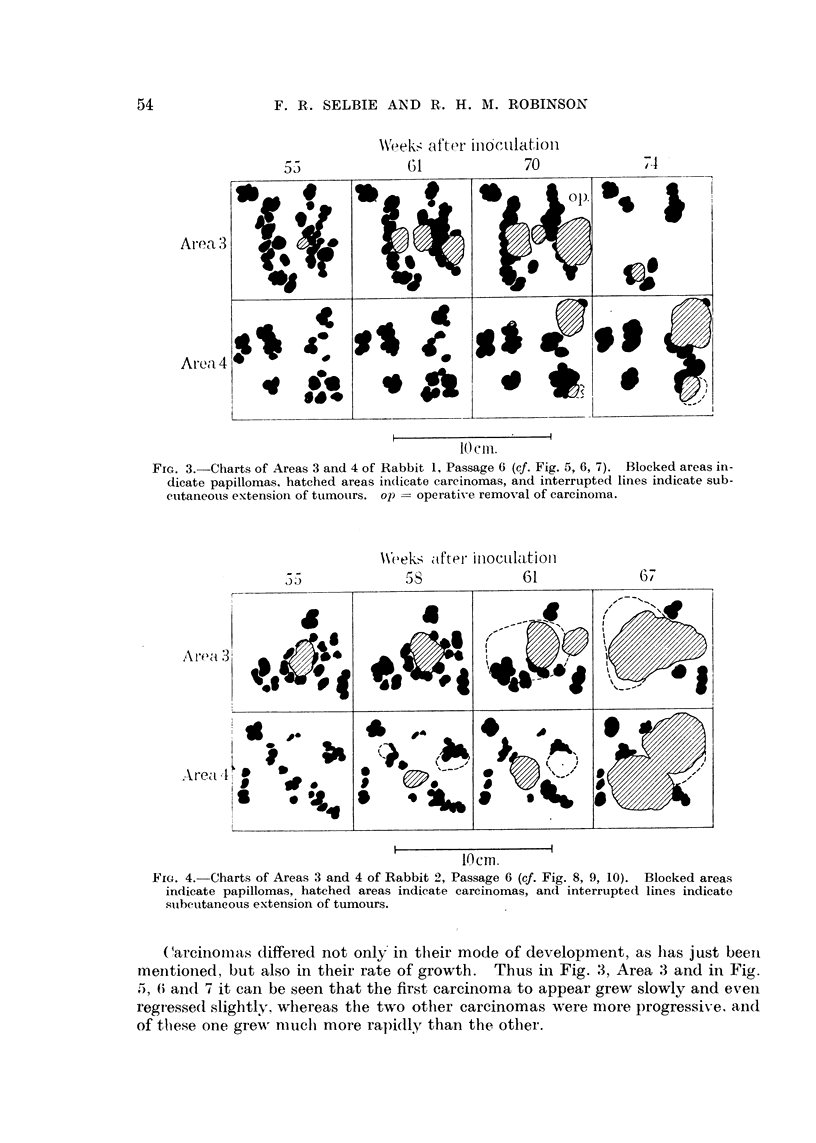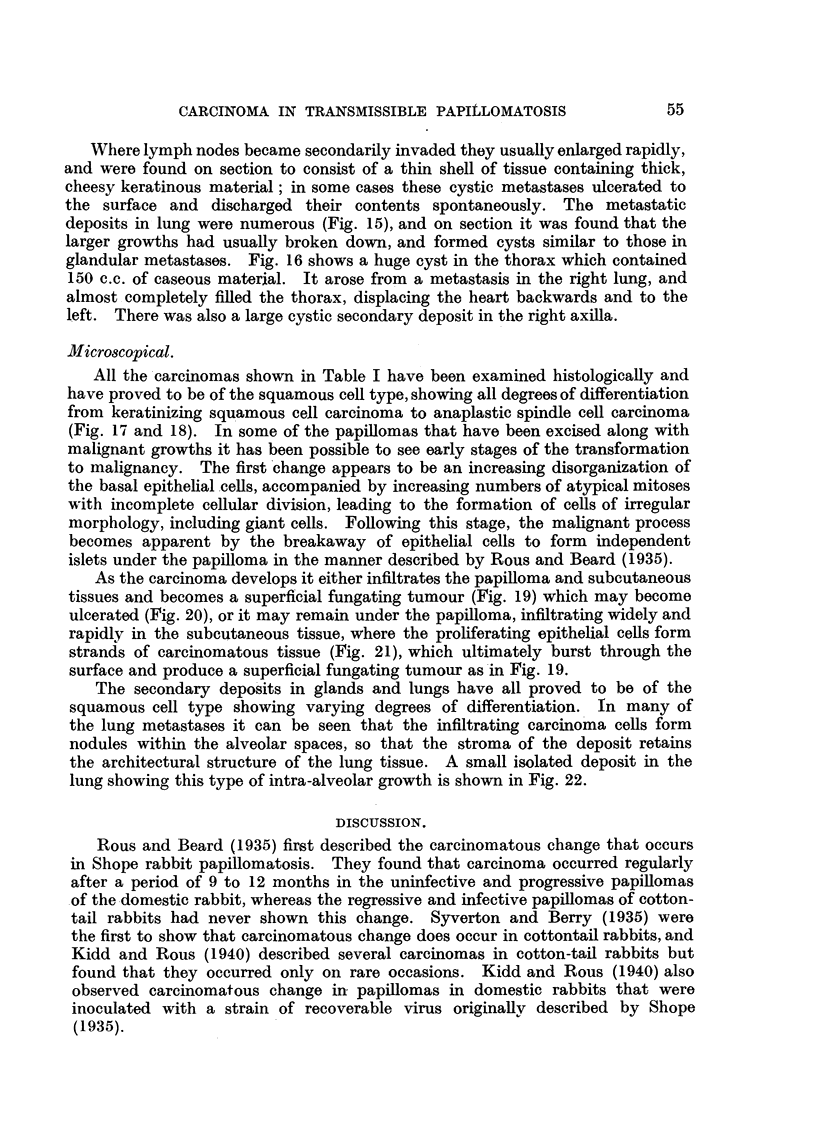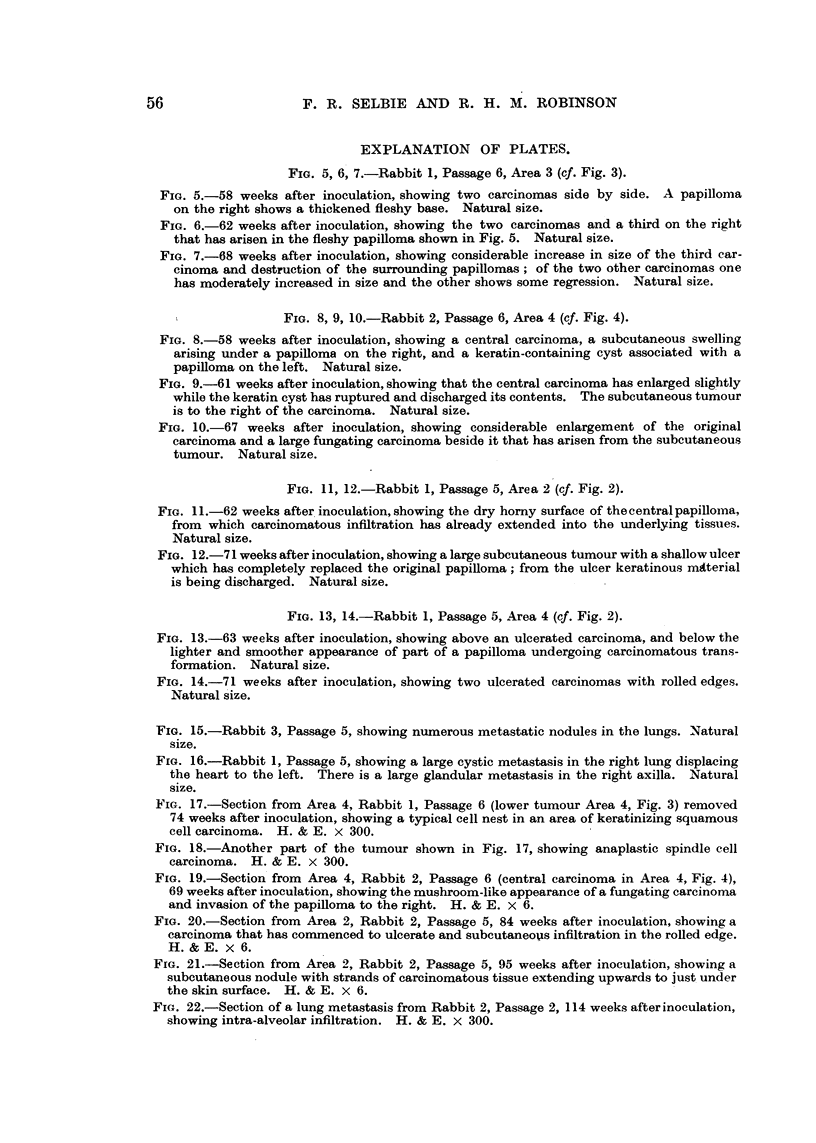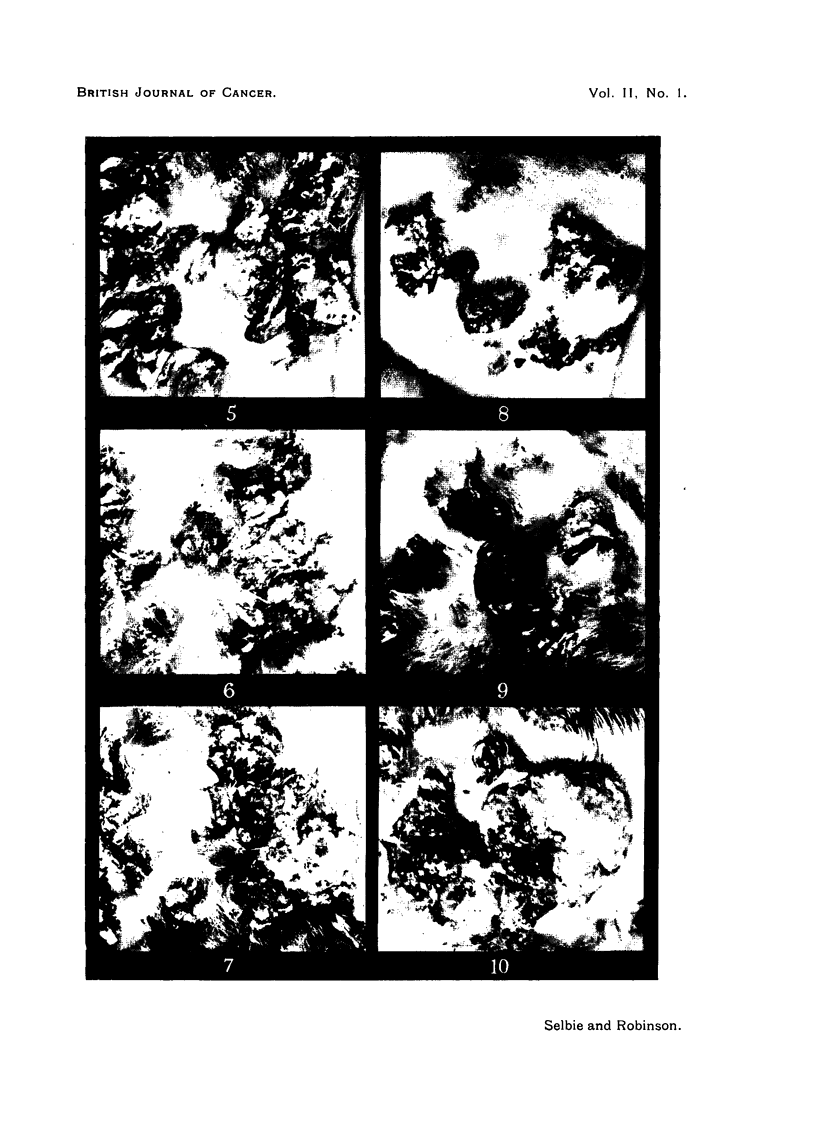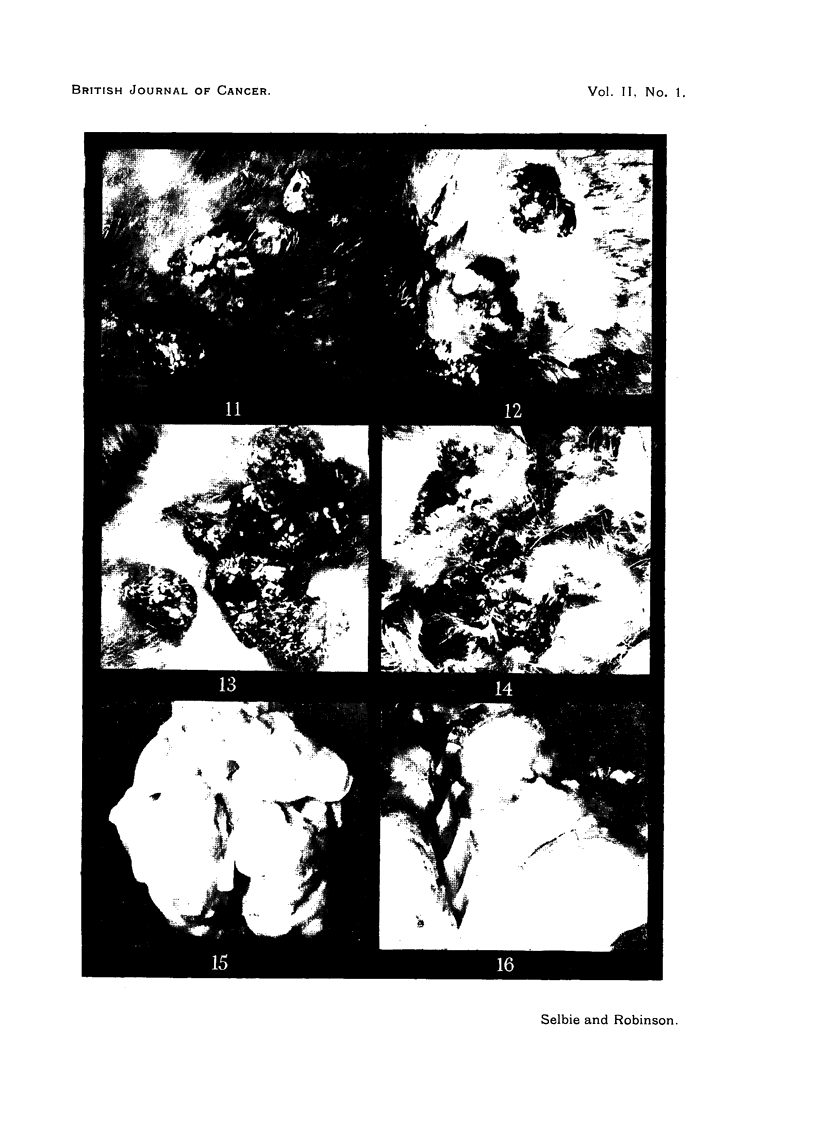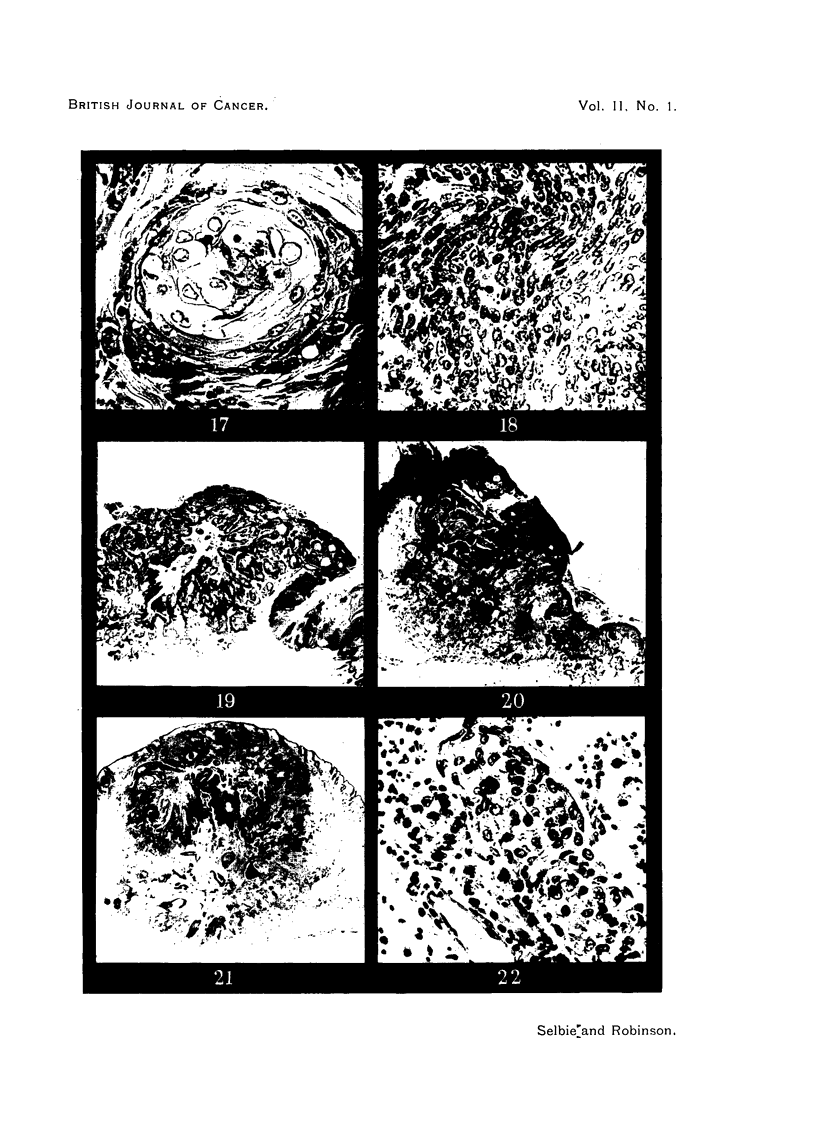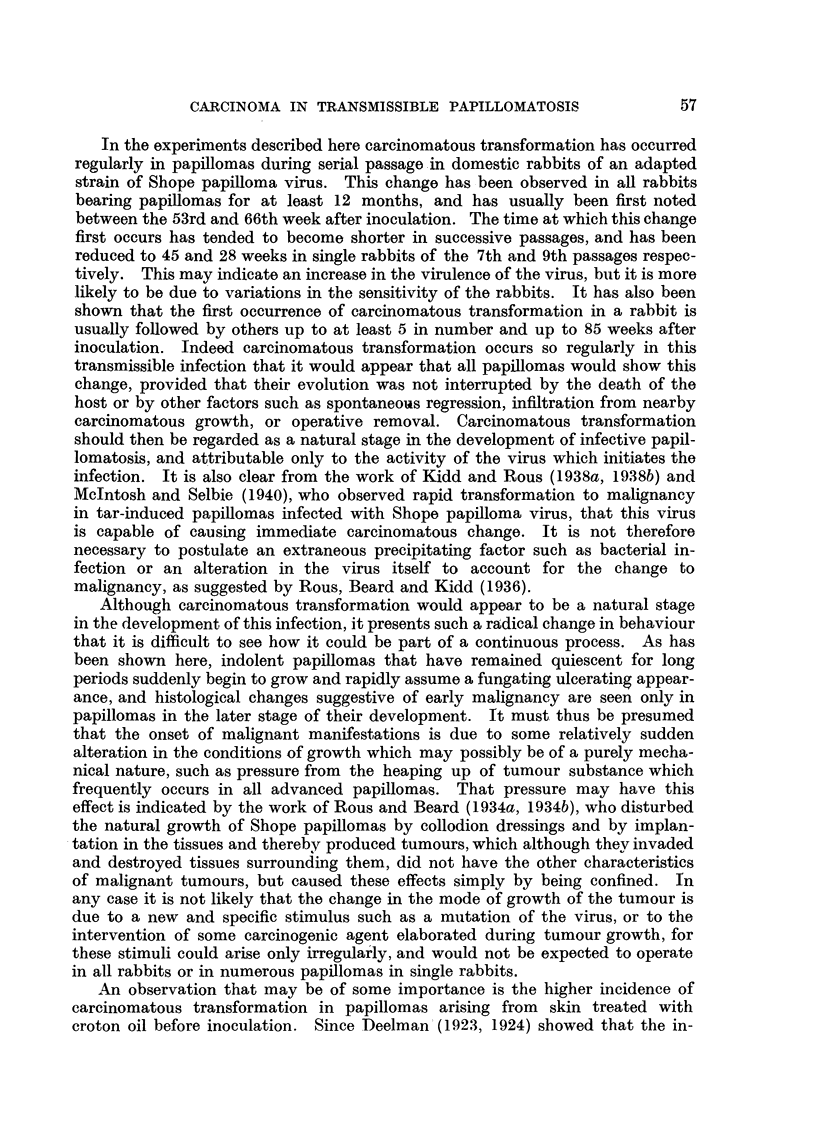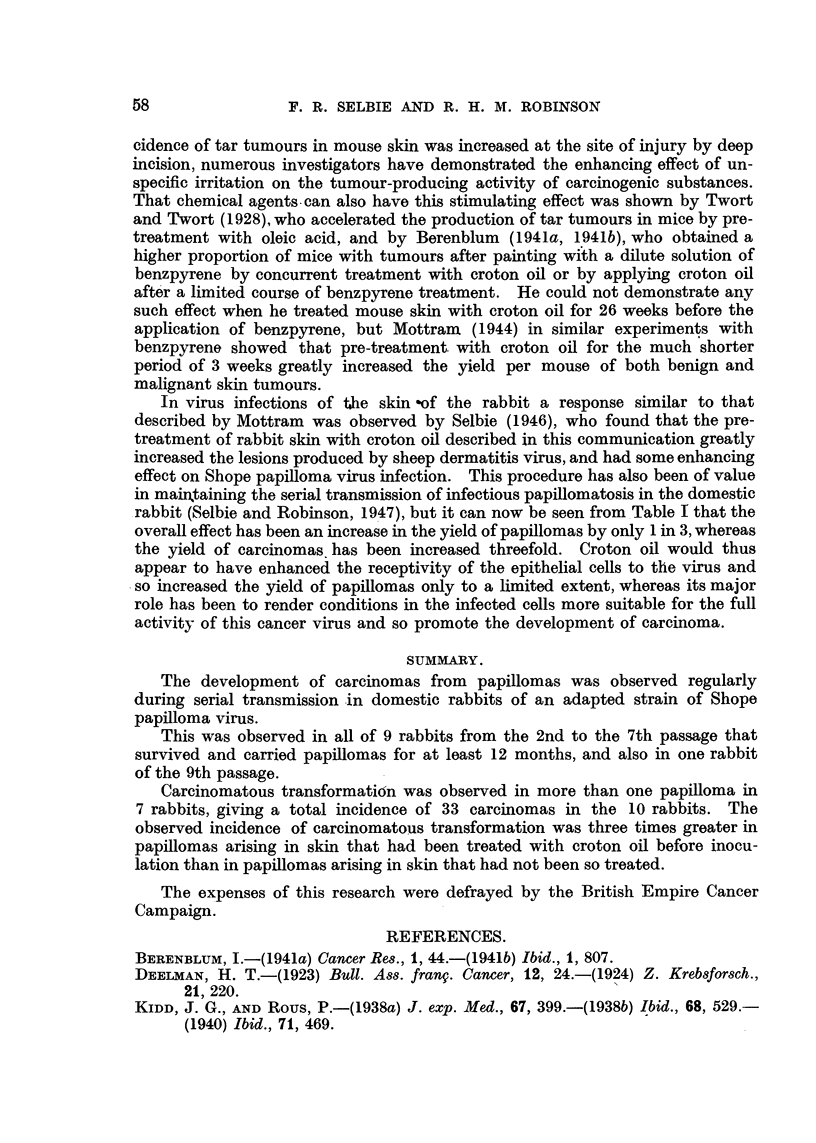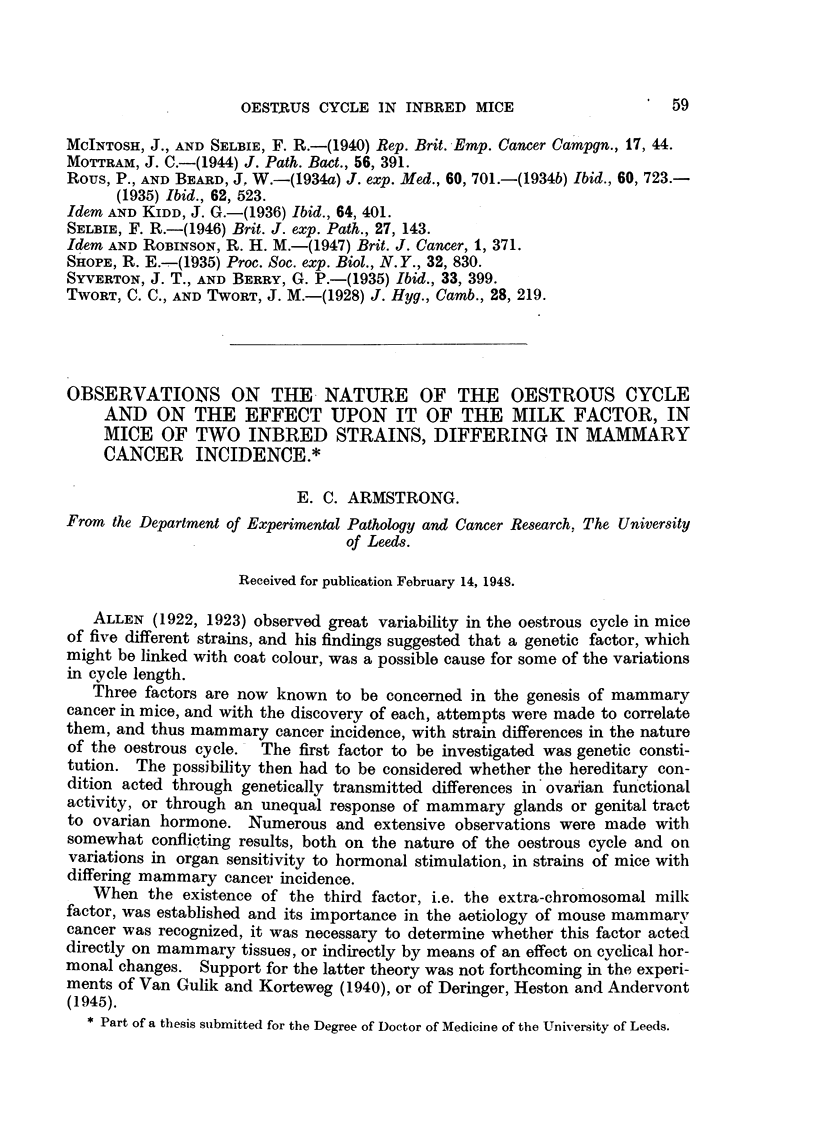# Incidence of Carcinoma in Transmissible Papillomatosis of the Domestic Rabbit

**DOI:** 10.1038/bjc.1948.7

**Published:** 1948-03

**Authors:** F. R. Selbie, R. H. M. Robinson

## Abstract

**Images:**


### INCIDENCE OF CARCINOMA IN TRANSMISSIBLE PAPILLOMATOSIS OF THE DOMESTIC RABBIT.

F. R. SELBIE AND R. H. M. ROBINSON.

*From the Bland-Sutton Institute of Pathology, Middlesex Hospital, London, W. 1.*

Received for publication February 23, 1948.

IN a previous communication we reported that a strain of the Shope rabbit papilloma had been transmitted for 10 serial passages in the domestic rabbit (Selbie and Robinson, 1947). The adaptation of this tumour virus as a transmissible infection in domestic rabbits occurred during experiments in which the simultaneous inoculation of Shope papilloma virus and sheep dermatitis virus in domestic rabbits produced papillomas that proved infective to other rabbits (Selbie, 1946). The further passage of this virus in domestic rabbits was materially helped by continuing the procedure of mixing a second virus suspension with the infective inoculum of papilloma virus, and also by treating the skin with croton oil before inoculation, but there was no evidence of an increase in the virulence of the virus suspensions during passage. In the present communication we shall show that carcinomatous transformation has occurred regularly in this transmissible papillomatosis, and in a manner similar to that found in the non-infective papillomatosis that is produced in domestic rabbits by the inoculation of extracts of papillomas from the cotton-tail rabbit, the original host of the Shope papilloma virus. We shall also discuss the implications arising from the incidence of carcinomatous transformation, and the influence thereon of the ancillary measures that have been used with a view to promoting infectivity.

### MATERIALS AND METHODS.

The rabbits to be considered here are those of Passages 2 to 7 which were alive and still carried papillomas 12 months after inoculation, and in addition Rabbit 2 of Passage 9, which has already developed several carcinomas, and died with extensive glandular metastases 40 weeks after inoculation. The early history of the rabbits comprising this series is shown in Table I of the earlier communication, where details are given of the methods used (Selbie and Robinson, 1947). In Passages 4 to 9, 4 areas of skin, 2 on either flank, were epilated by plucking out the fur, and 2 areas on the right side were treated with 6 applications on alternate days of 0·3 per cent croton oil in acetone before inoculation. The areas were inoculated following needle scarification with a 10 per cent extract of papillomas, the extract used on the posterior areas containing in addition 10 per cent of glycerolated sheep dermatitis lesions. Thus in all rabbits from Passage 4 in Table I, Area 1 represents normal skin inoculated with a 10 per cent extract of papillomas, Area 2, normal skin inoculated with a mixed extract of papillomas and sheep dermatitis lesions, Area 3, skin pre-treated with croton oil and inoculated with a 10 per cent extract of papillomas, and Area 4, skin pre-treated with croton oil and inoculated with the mixed virus suspensions. Rabbit 2, Passage 2 was inoculated with papilloma extract on normal skin only, and in Rabbit 4, Passage 3, the procedures for Areas 1 and 3 were omitted.

The rabbits were examined at weekly intervals from the 7th week onwards. The tumours were charted and their size was measured by callipers at regular intervals. As was noted in the earlier communication (Selbie and Robinson, 1947), there was a tendency for individual papillomas to unite during development, and in some instances papillomas regressed completely. Hence in this communication only those papillomas that were recognizable as single entities at 6 months will be considered. In areas with confluent or semi-confluent papillomatosis only a few papillomas could be distinguished as independent entities at 6 months, and no accurate count could be made, so that a fair estimate was given of 30 papillomas for confluent papillomatosis and 20 for semi-confluent papillomatosis. It became apparent early in this investigation that if the development of individual papillomas was to be studied over a prolonged period, cancers which arose in adjoining papillomas should be removed. Hence the entire carcinoma was widely excised as early as possible to prevent infiltration of surrounding papillomas (Fig. 3). In practice this was seldom possible to achieve, because at the same time it was necessary to observe the complete development of carcinoma with the formation of metastases.

### RESULTS.

### *Incidence of Carcinomatous Transformation.*

Table I shows the incidence of carcinomatous transformation in the individual areas of skin in all the rabbits in these experiments that survived for more than 12 months, and in Rabbit 2, Passage 9, which has already been mentioned. There are still 2 surviving rabbits in Passage 9 which, although they carry papillomas, have not yet, at 40 weeks, developed carcinomas and so have not been included. The carcinomas enumerated in Table I were confirmed histologically after being observed for some time as independent carcinomas arising from single papillomas. The estimate of the number of carcinomas arising on this basis is probably too low, particularly where the original papillomas were confluent or semi-confluent (Fig. 3 and 4). Thus in some instances carcinomatous change has been detected by histological examination in papillomas that appeared normal to the naked eye. Furthermore, it is possible that carcinomas occurring in two adjacent papillomas at approximately the same time would fuse so early as to make their identification as separate individuals unlikely. At present 5 of the 10 rabbits in Table I are alive, and are being kept

TABLE I.—*Number of Carcinomas Developing in Papillomas That Have Survived 6 Months.*

Treatment of inoculated skin and papilloma extract :—
Skin area 1 : No treatment of skin or extract.
,, ,, 2 : Papilloma extract mixed with extract of sheep dermatitis lesions.
,, ,, 3 : Pre-treatment of skin with croton oil.
,, ,, 4 : Treatments 2 and 3 combined.

| Passage No. | Rabbit No. | Area number. 1 | 2 | 3 | 4 | Period of observation in weeks. Died. | Alive. | Metastases. |
|---|---|---|---|---|---|---|---|---|
| 2 | 2 | 1/5 | .. | .. | .. | 114 | .. | Glands and lungs. |
| 4 | 4 | .. | 0/0 | .. | 2/13 | .. | 103 | Glands. |
| 5 | 1 | 1/1 | 1/3 | 0/9 | 2/9 | 91 | .. | Glands and lungs. |
| | 2 | 0/30 | 1/30 | 0/30 | 2/30 | .. | 95 | .. |
| | 3 | 0/0 | 1/30 | 3/8 | 1/4 | 77 | .. | Glands and lungs. |
| 6 | 1 | 0/2 | 0/2 | 4/20 | 2/20 | .. | 90 | .. |
| | 2 | 0/7 | 0/8 | 2/20 | 2/20 | 76 | .. | Glands. |
| 7 | 1 | 0/1 | 0/2 | 0/0 | 1/2 | .. | 66 | ,, |
| | 4 | 0/4 | 0/3 | 0/3 | 1/2 | .. | 66 | .. |
| 9 | 2 | 2/30 | 0/30 | 3/30 | 1/30 | 40 | .. | Glands. |
| Total | 10 | 4/80 | 3/108 | 12/120 | 14/130 | .. | .. | .. |

Numerator = Number of carcinomas.
Denominator = Number of papillomas present at 6 months.

for further observation along with rabbits from later passages. All still carry some papillomas with the exception of Rabbit 4, Passage 4, in which the papillomas have all been removed by operation.

It will be seen in Table I that carcinomatous transformation occurred in all the rabbits of Passages 2 to 7 that survived for at least 12 months, and also in one rabbit of Passage 9. It will also be seen that this change was observed in more than one site in 7 of the rabbits, and in as many as six in Rabbit 1, Passage 6 (Fig. 3), and in Rabbit 2, Passage 9, giving a total incidence of 33 carcinomas in the 10 rabbits. There was the expected tendency for more carcinomas to appear in rabbits with more papillomas, but a more significant factor is apparent from the total incidence in all passages of carcinomatous transformation in Areas 1, 2, 3 and 4, as shown in Table I. It will be seen that the sum of carcinomas that occurred in Areas 1 and 3 is similar to that in Areas 2 and 4, so that it is apparent that inoculation of a mixed suspension had no influence on the incidence of carcinomatous transformation. On the other hand, carcinomatous transformation occurred in only 7 of 188 papillomas in Areas 1 and 2 and in as many as 26 of 250 papillomas in Areas 3 and 4, where the skin was treated with croton oil before inoculation. It is thus evident that pre-treatment with croton oil increased the incidence of carcinomatous transformation in papillomas from about 1 in 27 to about 1 in 9·5, or approximately 3 times. This difference in incidence is just as great if it is based on the papillomas that were counted at 30 to 50 days, shown in Table I of the previous communication (Selbie and Robinson, 1947), in which case carcinomatous transformation occurred in 1 of 20 papillomas on skin pre-treated with croton oil and in 1 of 62 papillomas on untreated skin.

In Fig. 1 are shown the times at which carcinomatous growths were first detected in Passages 2 to 7, Passage 9 being omitted because the surviving rabbits have been observed for only 40 weeks after inoculation. It will be seen that the

FIG. 1.—Times of observations of carcinomas in all rabbits from the 2nd to the 7th passage. Each rabbit is represented by a line indicating the period of observation from the 40th week onwards. Blocked circles indicate carcinomas arising from papillomas on skin treated with croton oil before inoculation, and open circles indicate carcinomas arising from papillomas on untreated skin.
x= Removal by operation of all remaining papillomas. D = Died.

period before carcinomatous transformation was first observed tended to become shorter with successive passages, while in Rabbit 2 of Passage 9, which is not shown, this period was further reduced to 28 weeks. It is again shown here that the majority of carcinomas occurred in skin pre-treated with croton oil, but it is to be noted that carcinomatous transformation in treated skin was observed over a period of 40 weeks, between 45 and 85 weeks, whereas in normal skin carcinomas appeared only in the relatively short period of 13 weeks between 53 and 66 weeks. There was also an apparent delay in carcinomatous transformation in treated skin, for the mean times of appearance of carcinomas were 65 weeks on treated skin and 60 weeks on untreated skin. That this delay may be of some significance is shown in Passage 5, where there was a later development of carcinomas in treated skin than in untreated skin in all three rabbits. Taking the results as a whole, however, it can be seen that about half of the total yield of carcinomas on treated skin was obtained after the occurrence of carcinomatous transformation on untreated skin had ceased. Fig. 1 thus shows that treatment of the skin with croton oil not only increased the incidence of carcinomatous transformation, but prolonged the period over which this change was observed.

*Incidence of Metastases.*

It is shown in Table I that glandular metastases were observed in all the 5 rabbits that have died and in 2 of the 5 rabbits that are still alive. These metastases were found in the lymph nodes of the axilla and groin, and in 3 of the 7 rabbits more than one group of nodes was involved. The time at which glandular metastases were observed varied with the time at which the primary carcinomatous growth occurred, the limits being 33 weeks after inoculation in Rabbit 2, Passage 9, and 89 weeks in Rabbit 2, Passage 2, while the average time was 67 weeks for all the rabbits in the series. Rabbit 1, Passage 6 is interesting in that although 6 different primary cancers occurred between 55 and 71 weeks, no glandular metastases were observed up to the 90th week. It is not possible to draw any definite conclusions from this series concerning the incidence of glandular metastases as many of the primary growths were removed, and in consequence the opportunity for metastases to occur was reduced. Lung metastases were also found in 3 of the 5 rabbits that died. Two died as a result of operative procedures ; in Rabbit 2, Passage 6, there was cirrhosis of the liver with ascites, and Rabbit 2, Passage 9, was emaciated as a result of extensive glandular metastases. Metastases have not yet been observed at any site apart from lungs and lymph nodes.

*Development of Carcinoma.*

*Macroscopical.*

In our previous communication the development of the papillomas was fully described, and it was reported that after a period of quiescence or even retrogression, papillomas in some instances finally resumed rapid growth with the manifestations of malignancy (Selbie and Robinson, 1947). The earliest sign of malignant transformation was an increase in the diameter of the papilloma, with a concurrent thickening of the base. In Fig. 2, Area 2, the central papilloma had slowly enlarged from the time of its original appearance to attain a diameter of 1·4 cm. at 53 weeks. Nine weeks later it measured 1·8 cm. in diameter and the base had now thickened and was fleshy in appearance and consistency, in contrast to the dry horny surface shown in Fig. 11. Three weeks later, at 65 weeks, the diameter had increased to 2 cm., and ulceration of the surface had commenced as a result of carcinomatous infiltration. Another early sign of malignant transformation in papillomas was a pink rim of congestion surrounding the base where it merged with normal skin. The rabbits often gnawed these papillomas, presumably because of painful sensations arising from the infiltrating carcinoma, whereas ordinary papillomas rarely caused any sign of resentment. Palpation of such papillomas at this time frequently revealed that the undersurface was convex instead of flat or concave as is usual in quiescent papillomas.

When carcinomatous transformation became visible it usually commenced at the periphery of the papilloma and spread inwards, infiltrating the pigmented papilloma substance and replacing it with fleshy pink carcinomatous tissue. Simultaneously the invading growth spread under the skin surfaces, causing a typical heaped-up and rolled-edge appearance (Fig. 14). Finally the surface broke down, leaving an ulcer which readily became secondarily infected (Fig. 14). The usual type of carcinomatous transformation was superficial, and gave rise to the fungoid type of growth shown in Fig. 3, Areas 3 and 4, and in Fig. 7 and 10. In other cases, however, carcinomatous infiltration extended under the surface as in Fig. 2, Area 2, where the central papilloma at 62 weeks, although

Fig. 2.—Charts of Areas 1, 2 and 4 of Rabbit 1, Passage 5 (*cf.* Fig. 11, 12). Blocked areas indicate papillomas, hatched areas indicate carcinomas, and interrupted lines indicate subcutaneous extension of tumours. *op* = operative removal of carcinoma.

only beginning to show infiltration by carcinoma, was mounted on a mound of carcinomatous tissue (Fig. 11), and 9 weeks later presented the appearance shown in Fig. 12 of a shallow ulcer discharging the contents of keratinized cysts from a large subcutaneous carcinoma. Both types of development of carcinoma are shown in Fig. 4, Area 4 and Fig. 8, 9 and 10, where two tumours showed the first signs of carcinomatous transformation at the 57th week. One of these tumours developed directly into a fungating carcinoma, whereas the other formed a subcutaneous growth which broke through the skin after the 64th week, and finally produced a large fleshy mass similar to the first carcinoma. In many cases superficial carcinomas later showed deep extension in the subcutaneous tissues (Fig. 2, Area 4 ; Fig. 4, Area 3).

FIG. 3.—Charts of Areas 3 and 4 of Rabbit 1, Passage 6 (*cf.* Fig. 5, 6, 7). Blocked areas indicate papillomas, hatched areas indicate carcinomas, and interrupted lines indicate subcutaneous extension of tumours. *op* = operative removal of carcinoma.

FIG. 4.—Charts of Areas 3 and 4 of Rabbit 2, Passage 6 (*cf.* Fig. 8, 9, 10). Blocked areas indicate papillomas, hatched areas indicate carcinomas, and interrupted lines indicate subcutaneous extension of tumours.

Carcinomas differed not only in their mode of development, as has just been mentioned, but also in their rate of growth. Thus in Fig. 3, Area 3 and in Fig. 5, 6 and 7 it can be seen that the first carcinoma to appear grew slowly and even regressed slightly, whereas the two other carcinomas were more progressive, and of these one grew much more rapidly than the other.

Where lymph nodes became secondarily invaded they usually enlarged rapidly, and were found on section to consist of a thin shell of tissue containing thick, cheesy keratinous material; in some cases these cystic metastases ulcerated to the surface and discharged their contents spontaneously. The metastatic deposits in lung were numerous (Fig. 15), and on section it was found that the larger growths had usually broken down, and formed cysts similar to those in glandular metastases. Fig. 16 shows a huge cyst in the thorax which contained 150 c.c. of caseous material. It arose from a metastasis in the right lung, and almost completely filled the thorax, displacing the heart backwards and to the left. There was also a large cystic secondary deposit in the right axilla.

*Microscopical.*

All the carcinomas shown in Table I have been examined histologically and have proved to be of the squamous cell type, showing all degrees of differentiation from keratinizing squamous cell carcinoma to anaplastic spindle cell carcinoma (Fig. 17 and 18). In some of the papillomas that have been excised along with malignant growths it has been possible to see early stages of the transformation to malignancy. The first change appears to be an increasing disorganization of the basal epithelial cells, accompanied by increasing numbers of atypical mitoses with incomplete cellular division, leading to the formation of cells of irregular morphology, including giant cells. Following this stage, the malignant process becomes apparent by the breakaway of epithelial cells to form independent islets under the papilloma in the manner described by Rous and Beard (1935).

As the carcinoma develops it either infiltrates the papilloma and subcutaneous tissues and becomes a superficial fungating tumour (Fig. 19) which may become ulcerated (Fig. 20), or it may remain under the papilloma, infiltrating widely and rapidly in the subcutaneous tissue, where the proliferating epithelial cells form strands of carcinomatous tissue (Fig. 21), which ultimately burst through the surface and produce a superficial fungating tumour as in Fig. 19.

The secondary deposits in glands and lungs have all proved to be of the squamous cell type showing varying degrees of differentiation. In many of the lung metastases it can be seen that the infiltrating carcinoma cells form nodules within the alveolar spaces, so that the stroma of the deposit retains the architectural structure of the lung tissue. A small isolated deposit in the lung showing this type of intra-alveolar growth is shown in Fig. 22.

### DISCUSSION.

Rous and Beard (1935) first described the carcinomatous change that occurs in Shope rabbit papillomatosis. They found that carcinoma occurred regularly after a period of 9 to 12 months in the uninfective and progressive papillomas of the domestic rabbit, whereas the regressive and infective papillomas of cotton-tail rabbits had never shown this change. Syverton and Berry (1935) were the first to show that carcinomatous change does occur in cottontail rabbits, and Kidd and Rous (1940) described several carcinomas in cotton-tail rabbits but found that they occurred only on rare occasions. Kidd and Rous (1940) also observed carcinomatous change in papillomas in domestic rabbits that were inoculated with a strain of recoverable virus originally described by Shope (1935).

### EXPLANATION OF PLATES.

FIG. 5, 6, 7.—Rabbit 1, Passage 6, Area 3 (*cf.* Fig. 3).

FIG. 5.—58 weeks after inoculation, showing two carcinomas side by side. A papilloma on the right shows a thickened fleshy base. Natural size.

FIG. 6.—62 weeks after inoculation, showing the two carcinomas and a third on the right that has arisen in the fleshy papilloma shown in Fig. 5. Natural size.

FIG. 7.—68 weeks after inoculation, showing considerable increase in size of the third carcinoma and destruction of the surrounding papillomas ; of the two other carcinomas one has moderately increased in size and the other shows some regression. Natural size.

FIG. 8, 9, 10.—Rabbit 2, Passage 6, Area 4 (*cf.* Fig. 4).

FIG. 8.—58 weeks after inoculation, showing a central carcinoma, a subcutaneous swelling arising under a papilloma on the right, and a keratin-containing cyst associated with a papilloma on the left. Natural size.

FIG. 9.—61 weeks after inoculation, showing that the central carcinoma has enlarged slightly while the keratin cyst has ruptured and discharged its contents. The subcutaneous tumour is to the right of the carcinoma. Natural size.

FIG. 10.—67 weeks after inoculation, showing considerable enlargement of the original carcinoma and a large fungating carcinoma beside it that has arisen from the subcutaneous tumour. Natural size.

FIG. 11, 12.—Rabbit 1, Passage 5, Area 2 (*cf.* Fig. 2).

FIG. 11.—62 weeks after inoculation, showing the dry horny surface of the central papilloma, from which carcinomatous infiltration has already extended into the underlying tissues. Natural size.

FIG. 12.—71 weeks after inoculation, showing a large subcutaneous tumour with a shallow ulcer which has completely replaced the original papilloma ; from the ulcer keratinous material is being discharged. Natural size.

FIG. 13, 14.—Rabbit 1, Passage 5, Area 4 (*cf.* Fig. 2).

FIG. 13.—63 weeks after inoculation, showing above an ulcerated carcinoma, and below the lighter and smoother appearance of part of a papilloma undergoing carcinomatous transformation. Natural size.

FIG. 14.—71 weeks after inoculation, showing two ulcerated carcinomas with rolled edges. Natural size.

FIG. 15.—Rabbit 3, Passage 5, showing numerous metastatic nodules in the lungs. Natural size.

FIG. 16.—Rabbit 1, Passage 5, showing a large cystic metastasis in the right lung displacing the heart to the left. There is a large glandular metastasis in the right axilla. Natural size.

FIG. 17.—Section from Area 4, Rabbit 1, Passage 6 (lower tumour Area 4, Fig. 3) removed 74 weeks after inoculation, showing a typical cell nest in an area of keratinizing squamous cell carcinoma. H. & E. × 300.

FIG. 18.—Another part of the tumour shown in Fig. 17, showing anaplastic spindle cell carcinoma. H. & E. × 300.

FIG. 19.—Section from Area 4, Rabbit 2, Passage 6 (central carcinoma in Area 4, Fig. 4), 69 weeks after inoculation, showing the mushroom-like appearance of a fungating carcinoma and invasion of the papilloma to the right. H. & E. × 6.

FIG. 20.—Section from Area 2, Rabbit 2, Passage 5, 84 weeks after inoculation, showing a carcinoma that has commenced to ulcerate and subcutaneous infiltration in the rolled edge. H. & E. × 6.

FIG. 21.—Section from Area 2, Rabbit 2, Passage 5, 95 weeks after inoculation, showing a subcutaneous nodule with strands of carcinomatous tissue extending upwards to just under the skin surface. H. & E. × 6.

FIG. 22.—Section of a lung metastasis from Rabbit 2, Passage 2, 114 weeks after inoculation, showing intra-alveolar infiltration. H. & E. × 300.

5 8 6 9 7 10

Selbie and Robinson.

11

12

13

14

15

16

17

18

19

20

21

22

In the experiments described here carcinomatous transformation has occurred regularly in papillomas during serial passage in domestic rabbits of an adapted strain of Shope papilloma virus. This change has been observed in all rabbits bearing papillomas for at least 12 months, and has usually been first noted between the 53rd and 66th week after inoculation. The time at which this change first occurs has tended to become shorter in successive passages, and has been reduced to 45 and 28 weeks in single rabbits of the 7th and 9th passages respectively. This may indicate an increase in the virulence of the virus, but it is more likely to be due to variations in the sensitivity of the rabbits. It has also been shown that the first occurrence of carcinomatous transformation in a rabbit is usually followed by others up to at least 5 in number and up to 85 weeks after inoculation. Indeed carcinomatous transformation occurs so regularly in this transmissible infection that it would appear that all papillomas would show this change, provided that their evolution was not interrupted by the death of the host or by other factors such as spontaneous regression, infiltration from nearby carcinomatous growth, or operative removal. Carcinomatous transformation should then be regarded as a natural stage in the development of infective papillomatosis, and attributable only to the activity of the virus which initiates the infection. It is also clear from the work of Kidd and Rous (1938*a*, 1938*b*) and McIntosh and Selbie (1940), who observed rapid transformation to malignancy in tar-induced papillomas infected with Shope papilloma virus, that this virus is capable of causing immediate carcinomatous change. It is not therefore necessary to postulate an extraneous precipitating factor such as bacterial infection or an alteration in the virus itself to account for the change to malignancy, as suggested by Rous, Beard and Kidd (1936).

Although carcinomatous transformation would appear to be a natural stage in the development of this infection, it presents such a radical change in behaviour that it is difficult to see how it could be part of a continuous process. As has been shown here, indolent papillomas that have remained quiescent for long periods suddenly begin to grow and rapidly assume a fungating ulcerating appearance, and histological changes suggestive of early malignancy are seen only in papillomas in the later stage of their development. It must thus be presumed that the onset of malignant manifestations is due to some relatively sudden alteration in the conditions of growth which may possibly be of a purely mechanical nature, such as pressure from the heaping up of tumour substance which frequently occurs in all advanced papillomas. That pressure may have this effect is indicated by the work of Rous and Beard (1934*a*, 1934*b*), who disturbed the natural growth of Shope papillomas by collodion dressings and by implantation in the tissues and thereby produced tumours, which although they invaded and destroyed tissues surrounding them, did not have the other characteristics of malignant tumours, but caused these effects simply by being confined. In any case it is not likely that the change in the mode of growth of the tumour is due to a new and specific stimulus such as a mutation of the virus, or to the intervention of some carcinogenic agent elaborated during tumour growth, for these stimuli could arise only irregularly, and would not be expected to operate in all rabbits or in numerous papillomas in single rabbits.

An observation that may be of some importance is the higher incidence of carcinomatous transformation in papillomas arising from skin treated with croton oil before inoculation. Since Deelman (1923, 1924) showed that the incidence of tar tumours in mouse skin was increased at the site of injury by deep incision, numerous investigators have demonstrated the enhancing effect of unspecific irritation on the tumour-producing activity of carcinogenic substances. That chemical agents can also have this stimulating effect was shown by Twort and Twort (1928), who accelerated the production of tar tumours in mice by pre-treatment with oleic acid, and by Berenblum (1941*a*, 1941*b*), who obtained a higher proportion of mice with tumours after painting with a dilute solution of benzpyrene by concurrent treatment with croton oil or by applying croton oil after a limited course of benzpyrene treatment. He could not demonstrate any such effect when he treated mouse skin with croton oil for 26 weeks before the application of benzpyrene, but Mottram (1944) in similar experiments with benzpyrene showed that pre-treatment with croton oil for the much shorter period of 3 weeks greatly increased the yield per mouse of both benign and malignant skin tumours.

In virus infections of the skin of the rabbit a response similar to that described by Mottram was observed by Selbie (1946), who found that the pre-treatment of rabbit skin with croton oil described in this communication greatly increased the lesions produced by sheep dermatitis virus, and had some enhancing effect on Shope papilloma virus infection. This procedure has also been of value in maintaining the serial transmission of infectious papillomatosis in the domestic rabbit (Selbie and Robinson, 1947), but it can now be seen from Table I that the overall effect has been an increase in the yield of papillomas by only 1 in 3, whereas the yield of carcinomas has been increased threefold. Croton oil would thus appear to have enhanced the receptivity of the epithelial cells to the virus and so increased the yield of papillomas only to a limited extent, whereas its major role has been to render conditions in the infected cells more suitable for the full activity of this cancer virus and so promote the development of carcinoma.

### SUMMARY.

The development of carcinomas from papillomas was observed regularly during serial transmission in domestic rabbits of an adapted strain of Shope papilloma virus.

This was observed in all of 9 rabbits from the 2nd to the 7th passage that survived and carried papillomas for at least 12 months, and also in one rabbit of the 9th passage.

Carcinomatous transformation was observed in more than one papilloma in 7 rabbits, giving a total incidence of 33 carcinomas in the 10 rabbits. The observed incidence of carcinomatous transformation was three times greater in papillomas arising in skin that had been treated with croton oil before inoculation than in papillomas arising in skin that had not been so treated.

The expenses of this research were defrayed by the British Empire Cancer Campaign.

### REFERENCES.

BERENBLUM, I.—(1941*a*) *Cancer Res.*, **1**, 44.—(1941*b*) *Ibid.*, **1**, 807.

DEELMAN, H. T.—(1923) *Bull. Ass. franç. Cancer*, **12**, 24.—(1924) *Z. Krebsforsch.*, **21**, 220.

KIDD, J. G., AND ROUS, P.—(1938*a*) *J. exp. Med.*, **67**, 399.—(1938*b*) *Ibid.*, **68**, 529.—(1940) *Ibid.*, **71**, 469.

McIntosh, J., and Selbie, F. R.—(1940) *Rep. Brit. Emp. Cancer Campgn.*, **17**, 44.
Mottram, J. C.—(1944) *J. Path. Bact.*, **56**, 391.
Rous, P., and Beard, J. W.—(1934*a*) *J. exp. Med.*, **60**, 701.—(1934*b*) *Ibid.*, **60**, 723.—(1935) *Ibid.*, **62**, 523.
*Idem* and Kidd, J. G.—(1936) *Ibid.*, **64**, 401.
Selbie, F. R.—(1946) *Brit. J. exp. Path.*, **27**, 143.
*Idem* and Robinson, R. H. M.—(1947) *Brit. J. Cancer*, **1**, 371.
Shope, R. E.—(1935) *Proc. Soc. exp. Biol., N.Y.*, **32**, 830.
Syverton, J. T., and Berry, G. P.—(1935) *Ibid.*, **33**, 399.
Twort, C. C., and Twort, J. M.—(1928) *J. Hyg., Camb.*, **28**, 219.